# A Two-tiered compensatory response to loss of DNA repair modulates aging and stress response pathways

**DOI:** 10.18632/aging.100127

**Published:** 2010-03-29

**Authors:** Øyvind Fensgård, Henok Kassahun, Izabela Bombik, Torbjørn Rognes, Jessica Margareta Lindvall, Hilde Nilsen

**Affiliations:** ^1^ University of Oslo, The Biotechnology Centre, P.O. Box 1125 Blindern, 0317 Oslo, Norway.; ^2^ University of Oslo, Department of Informatics, P.O. Box 1080 Blindern, NO-0316 Oslo, Norway

**Keywords:** DNA repair, Caenorhabditis elegans, aging, gene expression profiling, Base Excision Repair, Nucleotide Excision Repair

## Abstract

Activation of oxidative stress-responses and downregulation of insulin-like signaling (ILS) is seen in Nucleotide Excision Repair (NER) deficient segmental progeroid mice. Evidence suggests that this is a survival response to persistent transcription-blocking DNA damage, although the relevant lesions have not been identified.  Here we show that loss of NTH-1, the only Base Excision Repair (BER) enzyme known to initiate repair of oxidative DNA damage inC. elegans, restores normal lifespan of the short-lived NER deficient xpa-1 mutant. Loss of NTH-1 leads to oxidative stress and global expression profile changes that involve upregulation of genes responding to endogenous stress and downregulation of ILS.  A similar, but more extensive, transcriptomic shift is observed in the xpa-1 mutant whereas loss of both NTH-1 and XPA-1 elicits a different profile with downregulation of Aurora-B and Polo-like kinase 1 signaling networks as well as DNA repair and DNA damage response genes. The restoration of normal lifespan and absence oxidative stress responses in nth-1;xpa-1 indicate that BER contributes to generate transcription blocking lesions from oxidative DNA damage.  Hence, our data strongly suggests that the DNA lesions relevant for aging are repair intermediates resulting from aberrant or attempted processing by BER of lesions normally repaired by NER.

## Introduction

The Base excision repair (BER) pathway is the main mechanism for removal of endogenously generated DNA base damage [1]. BER is initiated by DNA glycosylases that recognise and excise groups of related lesions [2]. There are at least 12 different mammalian DNA glycosylases, of which at least 7 have overlapping specificities towards oxidative DNA damage [3,4]. *Caenorhabditis elegans* (*C. elegans*) is a multicellular animal that encodes only two DNA-glycosylases: UNG-1 [5,6] and NTH-1 [7]. *C. elegans* is therefore an attractive system in which to study consequences of BER-deficiency in animals. Furthermore, the strong genetic and mechanistic correlation between stress resistance and longevity in *C. elegans* [8], allows us to probe the contribution of DNA damage, in particular oxidative DNA damage, and its repair to phenotypes associated with oxidative stress in large populations over the entire lifespan.

*C. elegans* NTH-1, a homolog of *E. coli nth*, was recently shown to have activity against oxidized pyrimidines [7]. A deletion mutant lacking exons 2 through 4, *nth-1(ok724),* is expected to be a null mutant and has elevated mutant rate [9] but no hypersensitivity to oxidizing agents [7]. The absence of a DNA-glycosylase with specificity towards oxidized purines in *C. elegans* is puzzling. Although *C. elegans* NTH-1 appears to have a weak ability to excise one of the major purine oxidation products (8-hydroxyguanine) [7], it seems likely that other DNA repair pathways such as Nucleotide Excision Repair (NER) might contribute to repair of oxidised purines in *C. elegans* as has been shown *in vitro* [10] and in *vivo* in *Saccharomyces cerevisiae* [11]. Genetic studies in *S. cerevisiae* show that NER is the preferred repair pathway for oxidative DNA damage in the absence of BER [12]. The NER pathway is highly conserved and orthologs of the core NER proteins are present in *C. elegans* [13]. XPA is required for formation of the preincision complex [14]. *C. elegansxpa-1* mutants are UV-sensitive [15,16] and the *xpa-1 (ok698)* mutant has reduced capacity to repair UV-induced DNA damage [13,17].

Expression profiling in NER-defective mice has revealed gene expression changes associated with segmental progeroid phenotypes [18-20]. For example, the NER-defective *Csbm/m/Xpa^-/-^* mice show suppression of signaling through the growth hormone (GH)/insulin growth factor 1 (IGF1) pathways and increased antioxidant responses. Similar changes could be induced in wild type mice through chronic administration of a reactive oxygen species (ROS) - inducing agent, suggesting that the transcriptional responses result from defects in transcription-coupled repair of oxidative DNA damage [21].

Although ROS are believed to be a main contributor to the stochastic endogenous DNA damage accumulating with increase age, and BER is the preferred pathway for repair of oxidative DNA damage, similar expression profiling has not been performed in BER defective animals. However, studies in *S. cerevisiae* suggest that mutants in BER as well as NER show global expression profile changes originating from unrepaired oxidative DNA damage after treatment with oxidizing agents [22,23].

Mutants in DNA glycosylases generally show very mild phenotypes, which has been attributed to the existence of backup enzymes with overlapping substrate specificities. Here we show that compensatory transcriptional responses contribute to maintain wild type phenotypes including lifespan, in the presence of endogenous oxidative stress in DNA repair mutants.

## Results

The transcriptional signatures of mixed populations of wild type N2 as well as *nth-1*, *xpa-1*, and *nth-1;xpa-1* mutants were measured using Affymetrix GeneChip *C.elegans* Genome Arrays in well fed animals cultured on plates to avoid stressful growth conditions.

### Oxidative stress response and reduced insulin/IGF-1 signaling in *nth-1(ok724)*

Since DNA damage responses often show small changes on the transcriptional level [24], we analysed the gene expression signatures using a fold-change cut-off criterion ≥1.8. We found a high number of differentially expressed transcripts between the N2 reference strain and the *nth-1* mutant considering the unstressed conditions of the animals: 2074 probe sets were differentially expressed ≥1.8 fold (Supplementary Table 1). The low number of transcripts regulated ≥4-fold (185 probe sets) suggests that there is a focused transcriptomic response to loss of the NTH-1 enzyme.

**Figure 1.**
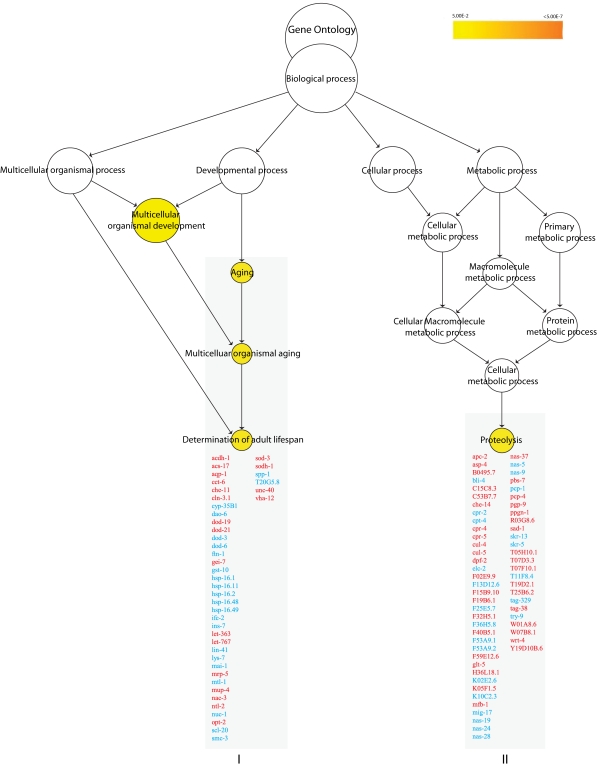
Overrepresented biological processes in N2 vs. * nth-1.* ** Enriched biological processes in *nth-1* vs. N2 are Aging *(p < 0.007)* and Proteolysis *(p < 0.01)*. The Aging cluster contains 17 genes involved in ILS signaling, including *ins-7* and *sod-3*. Genes responding to stress and steroid signaling are found in the Proteolysis cluster. Genes in red and blue are found to be upregulated and downregulated, respectively.

Gene ontology (GO) enrichment analysis revealed that genes involved in determining adult lifespan (*p< 0.007*) were enriched among the regulated genes in the *nth-1* mutant (Figure 1). Of these, 17 are known to act through the insulin/IGF-1 signaling (ILS) pathway. Reduced signaling through the canonical ILS pathway leads to nuclear localisation of the FOXO transcription factor DAF-16 [25]. A total of 84 genes previously identified as downstream targets of DAF-16 (*dod*) [26,27], were differentially regulated in *nth-1*, of which 67 were not assigned to the aging cluster based on present GO annotation. However, some confirmed targets of DAF-16 (e.g. *hsf-1, hsp-90, hsp-70*) were not differentially regulated, and there was no significant overlap between our dataset and the previously reported *daf-16* dataset [27] (data not shown). Moreover *dao-6*, which is positively regulated by DAF-16 and negatively regulated by DAF-2, was downregulated by 7.7-fold. Thus, the transcriptional changes in the *nth-1* mutant appear not to be dominated by DAF-16. The downregulation of *ins-1* and *ins-7* (2.17 and 3-fold, respectively), two DAF-2 agonists whose expression are repressed by DAF-16, likely reflects negative feedback inhibition of ILS rather than sensory neuronal input to the ILS pathway.

Previous genetic and genomic studies have demonstrated that there is a close interconnection between the ILS and stress-response pathways in *C. elegans* [8,28]. This is reflected in the*nth-1* dataset: The genes in the aging cluster, as well as individual genes regulated more than 4-fold (Supplementary Table 1), indicate that oxidative stress responses are activated. SOD-3 is a mitochondrial Mn-containing superoxide dismutase [29] and increased expression of *sod-3* has been reported in response to oxidative stress [30]. *sod-3* is a target of DAF-16, and the well established inverse regulation between *ins-7* and *sod-3* [31] is observed in *nth-1* (-3 and 1.84-fold, respectively). Activation of an oxidative stress response in *nth-1* is further suggested by the upregulation of *gst-4 *(2.31-fold), a regulator of SKN-1 which is a transcription factor mediating transcriptional responses to oxidative stress [32]. Regulation of steroid signaling and stress responses are also reflected in the second GO enriched cluster, proteolysis (*p < 0.01*) (Figure 1).

**Figure 2.**
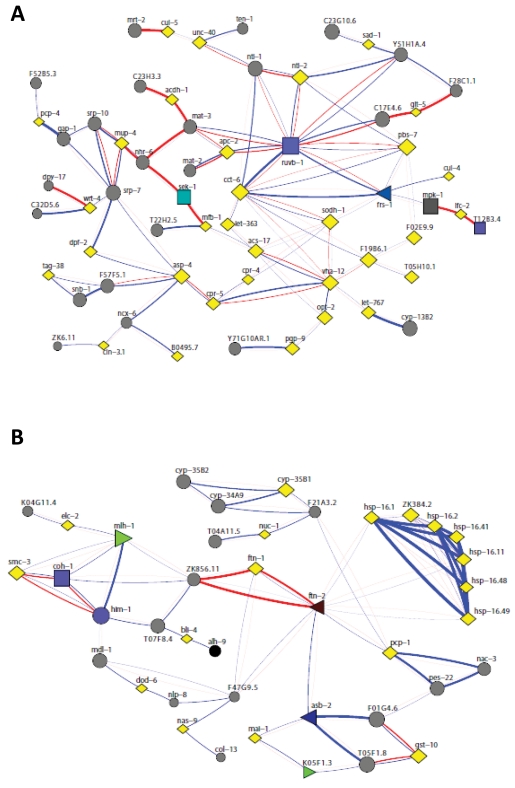
Network analysis revealed a close interconnection between the two enriched clusters in *nth-1.* (**A**) Functional interactions among upregulated genes in the two clusters was analysed using FunCoup [54]. A network of 97 most probable links between 95 genes was returned, involving 31 of the 58 regulated genes (12 and 19 from cluster I and II, respectively). (B) Network analysis of the 45 downregulated genes resulted in a network of 79 most probable links between 71 genes from both clusters.

A search for functional interactions among upregulated genes in the two clusters using the online functional interaction browser FunCoup revealed a close interconnection between the two enriched clusters involving 31 of the 58 regulated genes (12 and 19 from cluster I and II, respectively) (Figure 2A). The expression of the CeTOR (let-363) kinase is upregulated in *nth-1* (2.33-fold), possibly indicating activation of a survival response to stress. The TOR pathway controls protein homeostasis and contributes to longevity, and the network analysis indicates that TOR might connect the two clusters via the AAA+ ATPase homolog RUVB-1, a component of the TOR pathway [33]. Direct protein-protein interactions involving RUVB-1 have been demonstrated with several upregulated genes in both enriched GO clusters.

The protein-interaction network (Figure 2A) suggests that the transcriptional changes may also involve regulation of the redundant activities of the conserved p38 and JNK stress-activated protein kinase pathways: MFB-1, for example, directly interacts with SEK-1, a MAPK kinase required for germline stress-induced cell death independent of the CEP-1 (*C. elegans* p53) DNA damage response [34]. SEK-1 is also required for nuclear localisation of DAF-16 in response to oxidative stress [35]. It was suggested that oxidative stress mediates regulation of DAF-16 through activating the p38 signal transduction pathway upstream of DAF-16. Therefore, regulation of DAF-16 target genes in *nth-1* is consistent with activation of an oxidative stress response. Alternatively, the regulation of DAF-16 targets could be secondary to *aqp-1* upregulation. Aquaporin-1, a glycerol channel protein, was recently demonstrated to modulate expression of DAF-16-regulated genes and suggested to act as a feedback regulator in the ILS pathway [36]. There is a strong upregulation of *aqp-1* in *nth-1* (31-fold). Moreover, 6 out of 7 genes negatively regulated by AQP-1 are repressed in *nth-1* (Supplementary Table 2).

Network analysis of the 45 downregulated genes resulted in a network involving 71 genes from both clusters (Figure 2B). The pronounced downregulation of genes specifically responding to exogenous oxidative and heat stress (such as the *hsp-16* family, *ftn-1* and *gst-10*,*lys-7*, *mtl-1*) and anti-microbial immunity (several c-lectins, *cpr-2, ilys-3, abf-2, cnc-7*) in the *nth-1* mutant suggests that a specific response to endogenous stressors is triggered. Hence, loss of BER in *C. elegans* appears to induce transcriptional responses involving similar pathways as those regulated in mammalian NER mutants [19].

### Shared transcriptional responses in *nth-1* and *xpa-1* mutants

To experimentally validate whether there is similarity between the transcriptional programs associated with loss of BER and NER capacity in *C. elegans*, we collected the expression profile of the *xpa-1(ok698)* mutant. In *xpa-1*, we identified 2815 differentially expressed transcripts having a fold-change of ≥1.8 (Supplementary Table 3).

**Figure 3.**
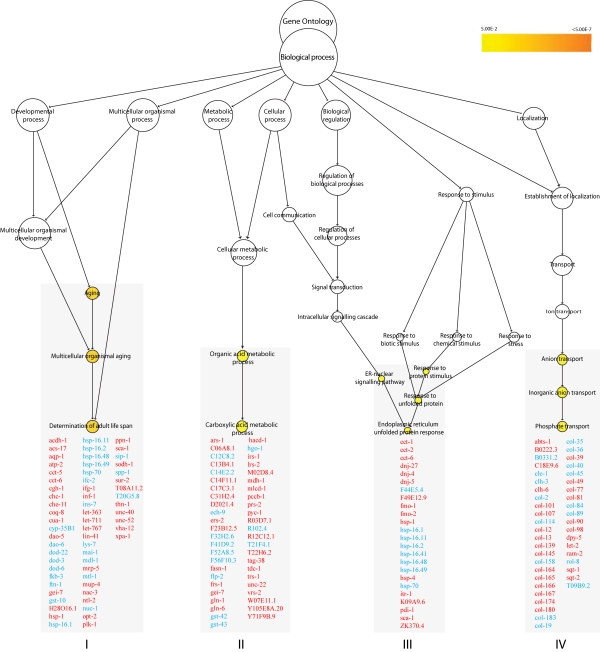
GO enrichment clusters in * xpa-1.* Genes that respond to oxidative stress and redox homeostasis are found in the enriched biological processes in *xpa-1* vs. N2. Aging *(p < 0.0007)*, regulation of carboxylic acid metabolism (*p < 0.03*), ER unfolded protein response (*p < 0.05*) and phosphate transport (*p < 0.02*). Genes in red and blue are found to be upregulated and downregulated, respectively.

**Figure 4.**
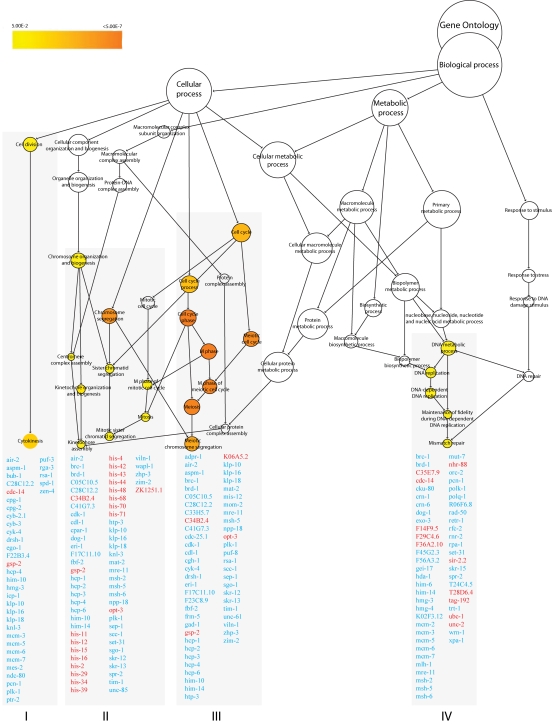
GO enrichment clusters in *nth-1;xpa-1*. The transcriptional response in the double mutant *nth-1;xpa-1* is dominated by genes involved in cell-cycle regulation (clusters I-III) and DNA repair (cluster IV). Cluster I (*p < 0.003*) and II (*p < 0.01*) contain genes that function in mitosis -related processes. Cluster III (*p < 0.00001*) reflect regulation of progression through meiosis. Genes involved in DNA repair and DNA damage checkpoint pathways in cluster IV (*p < 0.02*) are downregulated. Genes in red and blue are found to be upregulated and downregulated, respectively.

GO enrichment analysis revealed four significantly regulated clusters in *xpa-1* (Figure 3). The GO process determination of adult lifespan (*p < 0.0007*) was shared with *nth-1* (Figure 1), and 67% (28 out of 42) of the individual genes in this cluster in *nth-1* were shared with *xpa-1*. In *xpa-1*, genes that respond to oxidative stress and redox homeostasis are not only represented in the "aging" cluster, but are also found in the clusters containing genes involved in the ER unfolded protein response (*p < 0.05*) and regulation of carboxylic acid metabolism (*p < 0.03*). Network analysis of the 57 downregulated genes within the enriched clusters resulted in a network resembling that of nth-1 (Supplementary Figure 1). Thus, qualitatively similar responses were activated to compensate for the loss of NTH-1 or XPA-1. Regression analysis using the 1.8-fold-change data confirmed the similarity of the *nth-1* and expression profiles (R_2_ = 0.96). However, there is stronger modulation of gene expression in *xpa-1* compared to *nth-1*, with an increased number of transcripts with higher fold-change; e.g. expression of *ins-7* (8.8-and 3-fold), *aqp-1* (34.8- and 31-fold, respectively), and *hsp-16.49* (-15.99 and -5.58- fold) in *xpa-1* and *nth-1*, respectively (Supplementary Table 1 and 3).

### Somatic preservation in *nth-1;xpa-1*

Genes regulating adult lifespan were not among the four enriched GO processes identified from the 2787 regulated probe sets with fold-change ≥1.8 (1225 up and 1562 down) in *nth-1;xpa-1* (Figure 4 and Supplementary Table 4). Instead, the transcriptional response was dominated by genes involved in cell-cycle regulation (clusters I-III) and DNA repair (cluster IV). Cluster I (*p< 0.003*) and II (*p < 0.01*) contain genes that function in mitosis-related processes such as chromosome segregation, mitotic spindle assembly and stability, and replication licensing. Only 2 out of 36 genes in cluster I are upregulated. In contrast, 15 of the 64 genes present in cluster II are upregulated and most encode histone genes. Cluster III (*p < 0.00001*) share many genes with cluster I and II but reflect regulation of progression through meiosis.

Genes involved in DNA repair and DNA damage checkpoint pathways are enriched in Cluster IV (*p <0.02*). Naively, it could be expected that the double mutant would compensate for loss of integrity of two DNA repair pathways by upregulating alternative DNA repair modes. However, the opposite seems to be the case. Several mismatch repair, homologous recombination (HR), non-homologous end-joining (NHEJ), and DNA damage checkpoint genes, such as the *C. elegans* homolog of BRCA1 (*brc-1*) and its associated proteins, *brd-1* and *dog-1*, are downregulated (Table 1). Many uncharacterized genes that have previously been identified in screens for genes that result in mutator phenotypes when depleted by RNAi [37,38] were also suppressed in the *nth-1;xpa-1* mutant. Network analyses illustrate close interrelation of clusters I through IV also on protein level returning protein-protein interactions between 157 of the 212 genes in all clusters (data not shown).

### Suppression of the Aurora-B kinase and Polo-like kinase 1 regulatory network in *nth-1;xpa-1*

**Figure 5.**
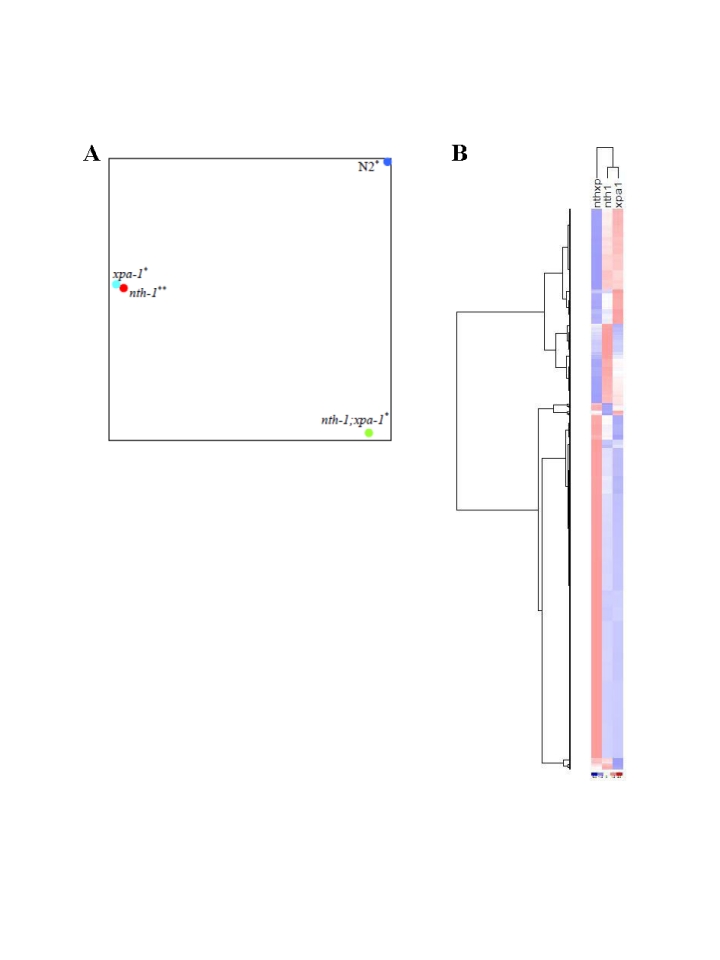
Comparative analyses of transcriptomes in DNA repair mutants. (**A**) The distance between respective mutants denoting the similarities or dissimilarities between *nth-1* (red circle), *xpa-1* (light blue circle), *nth-1;xpa-1* (green circle) and wild-type (blue circle) is shown using PCA. (**B**) Separation of the different mutant sample groups using Hierachical clustering.

The GO analysis suggests that the double mutant differs from either single mutant. Linear regression analysis comparing the overlapping transcripts in the ≥1.8-fold-change lists from *nth-1;xpa-1* and *xpa-1* confirmed this difference (R2 = 0.12) whereas the single mutants show significantly stronger correlation (R_2_ = 0.94). Principal Component Analysis (PCA) on the entire dataset (Figure 5A) confirms that the overall expression profiles of the single mutants cluster together and therefore resemble each other but, although *nth-1;xpa-1* clusters separately from the wild type, it seems to be in closer proximity to it than to either single mutant. Hierarchical clustering confirmed the closer relationship between *nth-1;xpa-1* and the wild type (Supplementary Figure 2). Hierarchical clustering of the mutants only revealed even more clearly that the single mutants are more similar to each other than either are to the double mutant. Several transcripts have opposite regulation, most notably in *xpa-1* and *nth-1;xpa-1* (Figure 5B). DNA repair and DNA damage response genes are prominent among the genes regulated in an opposite direction (a selection is presented in Table 1).

**Figure 6.**
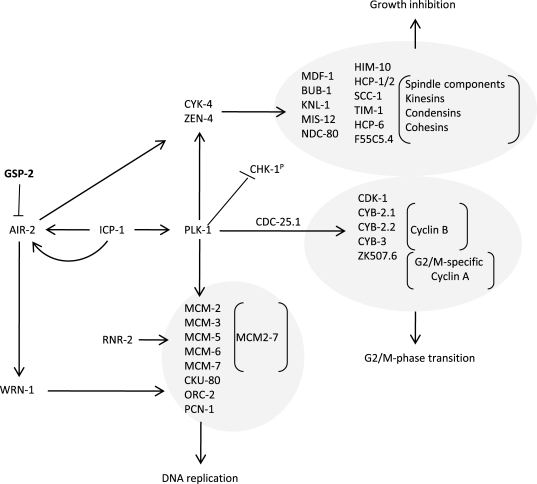
Somatic preservation through modulation of AIR-2 and PLK-1 signaling networks in * nth-1;xpa-1.* Genes encoding proteins known to stimulate AIR-2 and PLK-1 signaling are downregulated in* nth-1;xpa-1*: Plk-1 is known to stimulate activation of CDK-1, several cyclin B proteins and a G2/M specific cyclin A through CDC-25.1. Furthermore, PLK-1 and AIR-2 signaling coordinates cytokinesis and mitotic signaling at kinetochores via in the inner centromere ICP-1*, *regulates mitosis and cytokinesis through CYK-4 and ZEN-4, and could prevent firing of dormant replication origins via downregulation of MCM2-7. An inhibitor of AIR-2 activation, GSP-2*,* is one of the few upregulated genes**. **

Polo-like kinase 1 (PLK-1), which is upregulated in *xpa-1* (1.97-fold) but repressed in *nth-1;xpa-1* (-2.11-fold), has emerged as an important modulator of  DNA damage checkpoints [39,40]. Moreover, Aurora B kinase (*air-2*) is downregulated (-1.83-fold), and an inhibitor of AIR-2 activation, gsp-2, is one of the few upregulated genes in *nth-1;xpa-1*. Several other components of AIR-2 and PLK-1 networks are represented in the enriched GO clusters in the double mutant (Figure 4). Moreover, the transcriptional changes observed in *nth-1;xpa-1* involved several genes that are validated interactors of AIR-2 and PLK-1. The direction of the expression changes suggests that there is a concerted response that suppresses AIR-2 and PLK-1 signaling networks in the double mutant (Figure 6) that are consistent with published literature evidence: Plk-1 stimulates the activation of Cdk-1, several cyclin B proteins and a G2/M specific cyclin A through Cdc-25.1 [40]. The inner centromere protein (INCENP), ICP-1, coordinates cytokinesis and mitotic processes in the cell and integrates the PLK-1 and AIR-2 signaling at kinetochores. AIR-2 and PLK-1 regulate mitosis and cytokinesis through CYK-4 and ZEN-4 [41]. Downregulation of MCM2-7 could prevent firing of dormant replication origins which are often used when the transcriptional machinery is blocked or otherwise impaired [42]. Hence, the suppression of DNA metabolism suggested by the transcriptional signature reflects a concerted response. In summary, there seems to be a two-tiered compensatory response to loss of DNA repair in *C. elegans*: While lack of either BER or NER results in activation of genes responding to endogenous stressors and suppression of ILS, lack of both BER and NER shifts the transcriptional response to reduction of proliferation and somatic preservation through modulation of AIR-2 and PLK-1 signaling networks.

**Table 1.** Regulation of DNA repair and DNA damage response genes in DNA repair mutants*. * Gene classifications were determined based on previous analyses in references [54] and from information presented in Wormbase (www.wormbase.org). # A selection of DNA repair and DNA damage response genes regulated in *nth-1;xpa-1*. + Fold-changes calculated from the comparative analyses presented in Supplementary Table 1, 3 and 4.

pathway		Gene^#^	Fold-change^+^			
*nth-1*	*xpa-1*	*nth-1;xpa-1*	
**DNA repair**	BER/NER	*exo-3*			-2,38	
	MMR	*exo-1*			-2,03	
		*mlh-1*		2,83	-2,23	
		*msh-2*		1,83	-1,94	
		*msh-6*		2,33	-1,9	
	HR	*dna-2*		1,85	-2,43	
		*rad-50*			-1,85	
	NHEJ	*mre-11*			-1,94	
		*cku-80*			-2,1	
	DSB	*cnb-1*			2,19	
		*crn-1*			-1,93	
		*polk-1*			-2,21	
		*polq-1*		2,45	-2,61	
	Helicases	*dog-1*		1,89	-1,91	
		*him-6*		2,95	-2,15	
		*wrn-1*			-1,97	
	Other	*rpa-1*			-2,03	
		*pcn-1*			-2,24	
		*dpl-1*		2,06	-2,01	
		*rfc-2*			-1,97	
**DNA Damage Response/Cell Cycle**		*air-2*		2,09	-1,9	
		*ani-2*		1,82	-2,21	
		*brc-1*			-1,85	
		*brd-1*			-1,99	
		*C16C8.14*		1,94	2,16	
		*cdc-14*			-2,16	
		*cdc-25.1*			-1,88	
		*gst-5*			-2,26	
		*hil-1*		-2,08	2,47	
		*hsr-9*		1,83	-1,98	
		*K08F4.2*			-2,19	
		*lin-35*			-1,9	
		*mdf-1*			-1,96	
		*pme-5*	1,96	2,69	-1,95	

### Depletion of NTH-1 and XPA-1 induces oxidative stress responses

**Figure 7.**
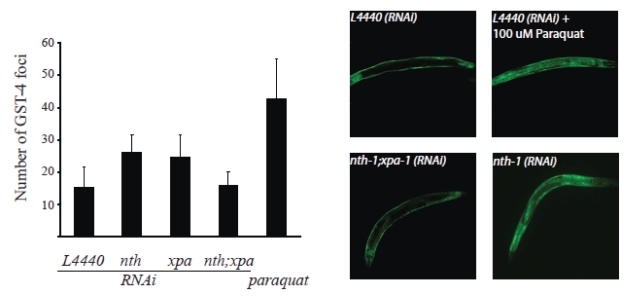
Oxidative stress is induced upon depletion of NTH-1 or XPA-1. The CL2166 reporter strain harbouring a GFP under the control of the *gst-4 *promoter was used to determine whether reduction of BER or NER, via *nth-1(RNAi)* and *xpa-1(RNAi) *respectively, or both pathways induces oxidative stress. A significant increase in GST-4 foci compared to the empty vector control (*L4440* (n = 97)) was observed in animals treated with RNAi against NTH-1 (n = 57) or XPA-1 (n = 95) *(p<0.0001)* using Student's *t*-test). Co-depletion of NTH-1 and XPA-1 (n= 46) did not give more GST-4 positive foci (*p = *0,507). GST-4 positive foci induced by paraquat (100 μM) was included as a positive control (n = 50).

Transcriptomic profiling strongly indicates that the *nth-1 *and *xpa-1* mutants experience oxidative stress. To experimentally validate whether loss of NTH-1 and XPA-1 induces oxidative stress, we took advantage of the established reporter strain CL2166, which expresses green fluorescent protein (GFP) under the control of the glutathione-S-transferase GST-4 promoter [32]. *gst-4* expression is upregulated in both *nth-1* and in *xpa-1* (2.31, and 2.01-fold respectively). Whereas GFP is normally expressed in hypodermal muscle, GFP-fluorescence increases in the body wall muscles and translocates to the intestinal nuclei upon oxidative stress As expected, paraquat, which generates superoxide *in vivo*, increases the average number of GFP positive intestinal nuclei up to 46 compared to 15 in untreated animals (*p < 0.001*). Depletion of NTH-1 or XPA-1 by RNAi significantly increased the number of foci to 26 and 25, respectively (*p < 0.001*) (Figure 7), thus demonstrating that even transient depletion of NTH-1 or XPA-1 induces oxidative stress responses. Codepletion of NTH-1 and XPA-1 did not increase the number of intestinal GFP-positive foci. The *gst-4*::GFP reporter assay therefore experimentally validated the high throughput genomic results and confirmed that loss of NTH-1 or XPA-1, but not both, leads to oxidative stress and activation of oxidative stress responses.

### The transcriptional changes do not protect against exogenous acute stress

**Figure 8.**
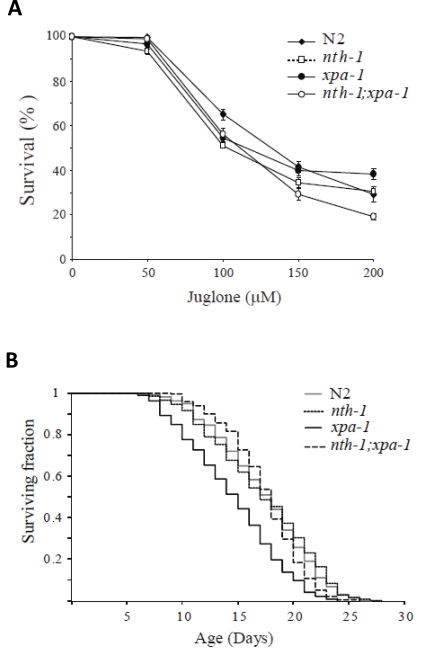
Compensatory responses specific for endogenous stressors. (**A**) Increase in the oxidative stress response do not confer resistance to juglone. Viability was scored as touch-provoked movement after 24 hour recovery from one hour exposure of young adults to juglone. Mean survival (+/- standard error of the mean) relative to untreated control was calculated from five independent experiments comprising a total of 250-350 animals.  (**B**) Lack of *nth-1* rescues the lifespan of an *xpa-1* mutant. Synchronized L4 larvae were placed on NGM plates at t = 0, incubated at 20°C, and transferred daily to fresh plates during the egg-laying period. The worms were monitored daily for touched-provoked movement; animals that failed to respond were considered dead. The *xpa-1* mutant shows a reduced lifespan compared to *nth-1* and *nth-1;xpa-1* and wild type, N2.

The expression profiling indicated that the transcriptional responses in the single-mutants are aimed at compensating for oxidative stress resulting from DNA-repair deficiency. The responses appear to be selectively tuned to compensate for endogenous stress. The down-regulation of other stress induced factors, such as the *hsp-16* family, may serve to prevent unsolicited activation of a full-blown stress response. Thus, we would not expect the DNA repair mutants to show resistance to oxidizing agents which is correlated with reduced ILS in *C. elegans* [8,30]. In agreement with previous reports, neither *xpa-1* [43], nth-1 [7] nor *nth-1;xpa-1* were hypersensitive to paraquat (data not shown). However, all mutants showed mild sensitivity to an acute exposure to another superoxide generating agent, juglone (Figure 8A) and mild heat-shock (data not shown). Hence, the upregulation of oxidative stress responses do not confer resistance to acute exogenous stress. These phenotypes are consistent with downregulation of genes responding to exogenous stressors as observed.

### Loss of NTH-1 restores normal lifespan in *xpa-1*

Reduced ILS induces longevity in *C. elegans* [44], but reduced ILS is also seen in segmental progeroid NER defective mice [21]. This apparent paradox can be interpreted as the reduced ILS in the DNA repair defective mice is part of a compensatory attempt to extend lifespan in organisms suffering from DNA damage associated stress. Thus, we were interested to test whether the reduced ILS in *nth-1* and *xpa-1* observed here was accompanied by reduced lifespan - or whether the compensatory response was sufficient to sustain normal lifespan. The lifespan of nth-1 was indistinguishable from the wild type, as was recently shown [7], whereas the *xpa-1* mutant displayed reduced lifespan compared to the wild type (mean survival of 14.5 and 17.3 days, respectively) (Figure 8B). In *C. elegans* therefore, as in mice, the challenges that loss of NER poses to the organism is more severe than loss of a single DNA-glycosylase. Our results demonstrate that this difference in challenge can be read out as a stronger activation of the antioxidant defense and reduction in ILS.

The *nth-1;xpa-1* double mutant has a more profound DNA repair defect and is expected to be unable to repair a much wider spectrum of DNA lesions. If the accumulation of DNA damage itself is the bigger lifespan reducing challenge in xpa-1, we would expect nth-1;xpa-1to be more severely affected. Interestingly, normal lifespan was restored in *nth-1;xpa-1* with a mean survival of 17.4 days. One possible interpretation of these results is that the oxidative lesions most relevant for aging are those that are normally repaired by NER, but are attempted processed by BER in the absence of the preferred repair pathway.

## Discussion

Mutants in DNA glycosylases generally have weak phenotypes. This has been explained by the existence of backup enzymes with overlapping substrate specificities. Here we present data that reveal additional explanations to how wild type phenotypes and lifespan are maintained in animals that lack a DNA glycosylase.

### Oxidative stress induced in DNA repair mutants

Here we present the first comprehensive report describing compensatory transcriptional responses to loss of base excision repair genes in animals. Using a well established transgenic reporter assay, we show that transient depletion of NTH-1 and XPA-1 by RNAi induces oxidative stress, thus it seems likely that this initiates transcriptome-modulation in the mutants. We show that lack of the NTH-1 and XPA-1 enzymes are accompanied by upregulation of oxidative stress responses tuned towards endogenous stressors. This is in agreement with identification of a focused compensatory response to BER intermediates (AP-sites and strand breaks) previously shown in *S.cerevisiae*, where the transcriptional responses differed from the common environmental stress response or the DNA damage signature [45]. A DNA-damage dependent ROS response to unrepaired oxidative DNA damage was previously demonstrated in *S. cerevisiae* BER and NER mutants [46]. Interestingly, no indication of oxidative stress or increased expression of oxidative stress response genes was observed in a mutant lacking both NTH-1 and XPA-1. This transcriptomic shift argues against a DNA-base damage dependent activation of oxidative stress responses, but instead indicates that the DNA repair enzymes mediate signaling to activate stress response pathways. Although the upstream signaling events in the *nth-1;xpa-1* double mutant remain to be elucidated, the modulation of AIR-2 and PLK-1 interaction networks may be a consequence of absence of DNA repair enzyme-mediated signaling of transcription blocking lesions. Alternatively, the extensive new synthesis of histone genes suggests that signaling involves chromatin dynamics in the absence of the global genome damage binding proteins, NTH-1 and XPA-1.

The biological significance of transcriptome modulation seen here is confirmed by the DNA repair mutants showing a mild sensitivity to oxidizing agents. It seems likely that the downregulation of the *hsp-16* family and *ftn-1* contributes to the higher sensitivity to juglone in *xpa-1* and *nth-1*, particularly as it is unlikely that a short acute exposure to oxidizing agents (or heat-stress) may lead to DNA-damage mediated toxicity on organismal level.

### Conserved compensatory responses to BER and NER deficiency

Few systematic studies have been performed to look at transcriptomic changes in BER mutant animals and none, to the best of our knowledge, have compared mutants in BER and NER.

A study on gene expression profiling in BER- or NER-defective mutant *S. cerevisiae* showed transcriptional changes in mutants defective in both pathways after treatment with hydrogen peroxide [22], but not in the double mutant. Instead, transcriptome changes in the BER/NER defective strain were already elicited from unrepaired spontaneous DNA damage [23]. To test the generality of our finding, we re-analyzed the baseline data sets from *S. cerevisiae* BER (Ntg1, Ntg2, and Apn1 deficient), NER (Rad1 deficient) and BER/NER defective mutants. We performed GO enrichment analysis on expressed transcripts in the individual strains (Supplementary Table 5). This analysis showed that BER-defective cells had few expressed transcripts and only one enriched GO process, DNA replication (*p< 0.05*). Informative enriched GO processes found only in the NER defective strain include transcription regulation, ubiquitin dependent protein degradation, sister chromatid segregation, and cell communication (*p < 0.01*). The BER/NER and NER defective cells share many enriched GO clusters and show 38% overlap of individual expressed transcripts. Enriched GO processes found only in the BER/NER mutant include DNA repair, DNA packaging, response to DNA damage stimulus, and cell-cycle checkpoint. Regulation of RNA polymerase II transcription was not enriched in the BER/NER mutant. Therefore, the main conclusions drawn here from BER, NER and BER/NER deficient *C. elegans*, resemble those previously seen in *S. cerevisiae* [22,23,45]: i) BER mutants show transcriptomic changes. ii) Loss of NER induces more substantial transcriptional responses than BER involving modulation of RNA metabolism and regulation of transcription iii) Loss of BER in the NER mutant shifts the response to regulate processes that maintain DNA integrity.

### Reduced mean lifespan in *xpa-1(ok698)*

The first *xpa-1* mutant identified,*rad-3(mn159)*, was reported to have a near-normal lifespan [15] but there are conflicting reports on the lifespan of *xpa-1(ok 698)* allele ranging from normal [43] to a maximum lifespan of 15 days compared to 25 days in the wild type [17]. Here, we show a moderate reduction of lifespan in *xpa-1*. We did therefore not anticipate that the XPA-1 mutant would display a transcriptional profile resembling that of the segmental progeroid NER defective mice [19]. Nevertheless, the reduced lifespan is entirely consistent with the transcriptional changes observed. A recent transcriptomic signature of *Xpa^-/-^* mouse dermal fibroblasts shows that suppression of ILS and activation of oxidative stress responses is also seen in DNA repair mutants that do not exhibit accelerated aging [47]. In support of this, GO enrichment analysis, performed as part of the present study, on the differentially expressed genes in *Xpa^-/-^* mice [21] showed enrichment of genes that regulate lifespan. Hence, the transcriptomic changes in NER mutants are conserved.

However, the consequences of loss of XPA-1 appear more severe in *C. elegans* compared to mice, both with respect to transcriptional regulation and lifespan. This might indicate that *C. elegans* XPA-1 contributes to repair of spontaneous DNA damage. The 13-fold elevated mutation accumulation rate in *xpa-1* compared to 7-fold in *nth-1* [9], supports this possibility.

## Concluding remarks

Based on a large body of evidence indicating that persistent transcription-blocking DNA damage cause attenuation of ILS and activation of oxidative stress responses [18-20,47], it is reasonable to speculate that the transcriptome modulation in the *xpa-1* mutant reflects accumulation of transcription blocking DNA lesions. That similar changes are seen in the *nth-1* mutant suggests that such transcriptomic shifts may be a general strategy for survival in DNA repair mutants. Since BER is the main pathway for repair of endogenous oxidative lesions, this lends further support to the notion that oxidative DNA damage contributes to these phenotypes. However, few known BER substrates are recognized as being transcription blocking and cyclopurines, that are often mentioned in this context [47], are NER substrates [48]. The qualitatively different responses in the double mutant support a model where the NTH-1 and XPA-1 enzymes themselves take part in the signaling events that result in activation of responses tunes to compensate for endogenous stress and suppression of ILS. We hypothesize that binding or inefficient processing of oxidative damage relevant to aging in the absence of the preferred repair pathway leads to formation of transcription blocking structures or signaling inter-mediates. The restoration of normal lifespan upon deletion of NTH-1 supports this hypothesis and strongly suggests that inefficient BER-mediated processing of lesions normally repaired by NER, results in intermediates that pose a lifespan-reducing challenge.

## Materials and methods

Strains and culture conditions. All strains were maintained at 20^o^C as described [49].  The wild-type Bristol N2, *nth-1(ok724)*, *xpa-1(ok698)* and the transgenic strain CL2166 (*dvIs19[pAF15(gst-4::GFP::NLS*) were all kindly provided by the Caenorhabditis Genetic Center (University of Minnesota, St Paul, MN, USA). The double mutant *nth-1(ok724)*; *xpa-1(ok698)* was generated for this work. All strains were backcrossed 3-4 times immediately ahead of the experiments.

RNA isolation and microarray processing. Mixed stage populations of N2, *xpa-1*, *nth-1* and *nth1;xpa-1* were reared at 20°C on HT115(DE3)-seeded on NGM plates (30 plates per replicate, 3 replicates per strain) until the nematodes had cleared the plates of food.  Worms were washed off with S-medium, left to digest remaining food in the gut, and washed 3 times before pelleting and suspended in TRIZOL and frozen at -80°C.  Total RNA isolation was then performed by standard procedures (Invitrogen). Synthesis of double stranded cDNA and Biotin-labeled cRNA was performed according to manufacturer's instructions (Affymetrix, Santa Clara, CA, US). Fragmented cRNA preparations were hybridized to the Affymetrix GeneChip *C. elegans* Genome Arrays on an Affymetrix Fluidics station 450. Data deposit footnote: GSE16405.

Data and statistical analysis. The processing and primary data analysis was performed in DNA-Chip Analyzer (dChip) (http://biosun1.harvard.edu/complab/dchip/) where normalization (invariant set), model-based expression correction (PM-only model), comparative analysis, PCA and Hierachical clustering was conducted. XLStat (Excel) was used for linear regression analysis. Enriched GO clusters were analysed using Cytoscape [50], in conjunction with the plug-in system BiNGO [51] in addition to DAVID (http://niaid.abcc.ncifcrf.gov) [52,53]. The Hyper-geometric Test with Benjamini-Hochberg False Discovery Rate Correction was chosen for both the analyses [51]. Functional interaction networks were generated using the online browser FunCoup [54].

*gst-4 *::GFP  expression. RNAi feeding constructs in the pL4440 vector harbouring the NTH-1 and XPA-1 open reading frames were generated by Gateway Technology and transformed into *E. coli*  HT115(DE3). NGM plates containing 2 mM IPTG seeded with bacteria expressing the empty vector control L4440 or *nth-1*(*RNAi),xpa-1(RNAi)* individually or in combination were activated at 37°C for one hour and left to cool to room temp before the CL2166 reporter strain was added.  Plates containing 100 μM paraquat (Sigma) were used as positive control. All plates were incubated at 20°C for 2 days before quantification of GST-4 foci on a Nikon eclipse Ti microscope.

Sensitivity to oxidising agents. The sensitivity to the superoxide-generating compound juglone (Sigma) was performed as previously described [55]. Briefly, young adults were exposed to juglone dissolved in M9 buffer for 1 hour in liquid culture. Viability was scored as touch-provoked movement after a 24h recovery period at 20**°**C on NGM plates seeded with OP50.

Lifespan determination. Assessment of lifespan was performed essentially as described [56].  Briefly, synchronized L4 larvae were placed on NGM plates at t = 0, incubated at 20**°**C, and transferred daily to fresh plates during the egg-laying period. The worms were monitored daily for touched-provoked movement. Triplicates comprising 10 plates containing at least 10 worms per plate were performed for each strain. Kaplan-Meier survival distributions were generated and Wilcoxon's log rank test was used to assess significance.

Comparisons with published microarray and Real-Time PCR data. Our datasets were compared to data from van der Pluijm et al. [21]: Significantly differentially expressed transcripts found in *Xpa^-/-^* compared to wild type mice were extracted and translated into corresponding *C. elegans* orthologs (using NetAffx, http://www.affymetrix.com/analysis/index.affx). These orthologous set of genes were analysed using Cytoscape [50] to find enriched GO Biological Processes.

Next, we compared our results to datasets generated by  Evert et al. from untreated wild type, BER, NER and BER/NER *S. cerevisiae* mutants [51]. Cytoscape was used in order to get a comprehensive overview of enriched Biological Processes in each individual sample group. Also, using dChip, expressed transcrips from each sample group were re-analysed in a comparative analysis giving a list of differentially expressed transcripts with a fold-change ≥2 between wild type and mutant cells. In dChip, replicates were combined and a mean signal value was calculated prior to the comparative analysis. These fold-change lists were then imported into Cytoscape for GO enrichment analysis.

Finally, we extracted genes found to be significantly differentially expressed in the *aqp-1* compared to the wild type in a Real-Time PCR data set from a recent paper by Lee et al. [51] and compared these to our data set.

## Supplementary data

Supplementary Figure 1 Network analysis of downregulated genes within the enriched GO clusters in *xpa-1p.*

Supplementary Figure 2 Hierarchical clustering between the transcriptomic profiles of N2, */nth-1,/ /xpa-1/*, and */nth-1;xpa-1/.*

Supplementary Table 1 Comparative results from wild type N2 vs *nth-1*.

Supplementary Table 2 Overlapping genes in *aqp-1* and *nth-1* and *xpa-1.*

Supplementary Table 3 Comparative results for wild type N2 vs. *xpa-1.*

Supplementary Table 4 Comparative results for wild type N2 vs. *nth-1;xpa-1.*

Supplementary Table 5 Gene Ontology classes enriched in BER mutant from Evert et al. 2004. Gene Ontology classes enriched in NER mutant from Evert et al. 2004. Gene Ontology classes enriched in BERNER mutant from Evert et al. 2004. Gene Ontology enriched classes unique to each mutant from Evert et al. 2004.
