# Supplementary figures and images for: A Two-tiered compensatory response to loss of DNA repair modulates aging and stress response pathways
Source: Aging (Albany NY). 2010 Mar 29;2(3):133–59. doi: 10.18632/aging.100127 (PMC2871243; doi:10.18632/aging.100127)

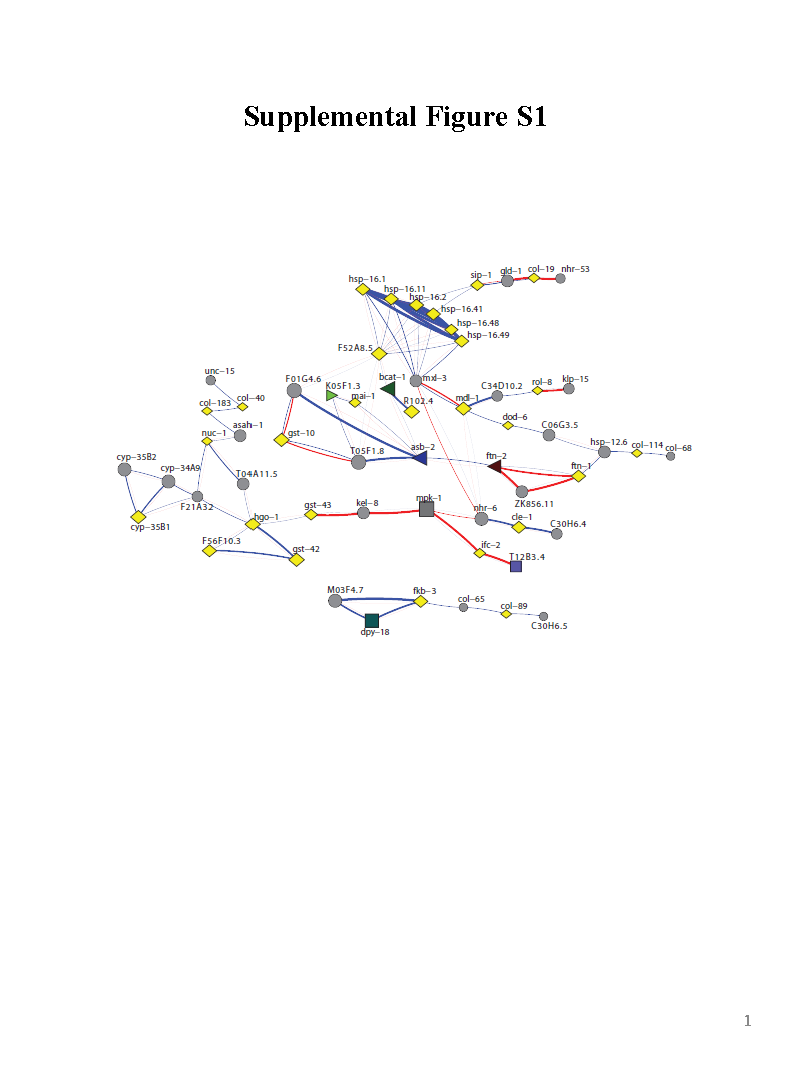

Supplement: Supplementary Figure 1 [file aging-02-133-s001.tif]

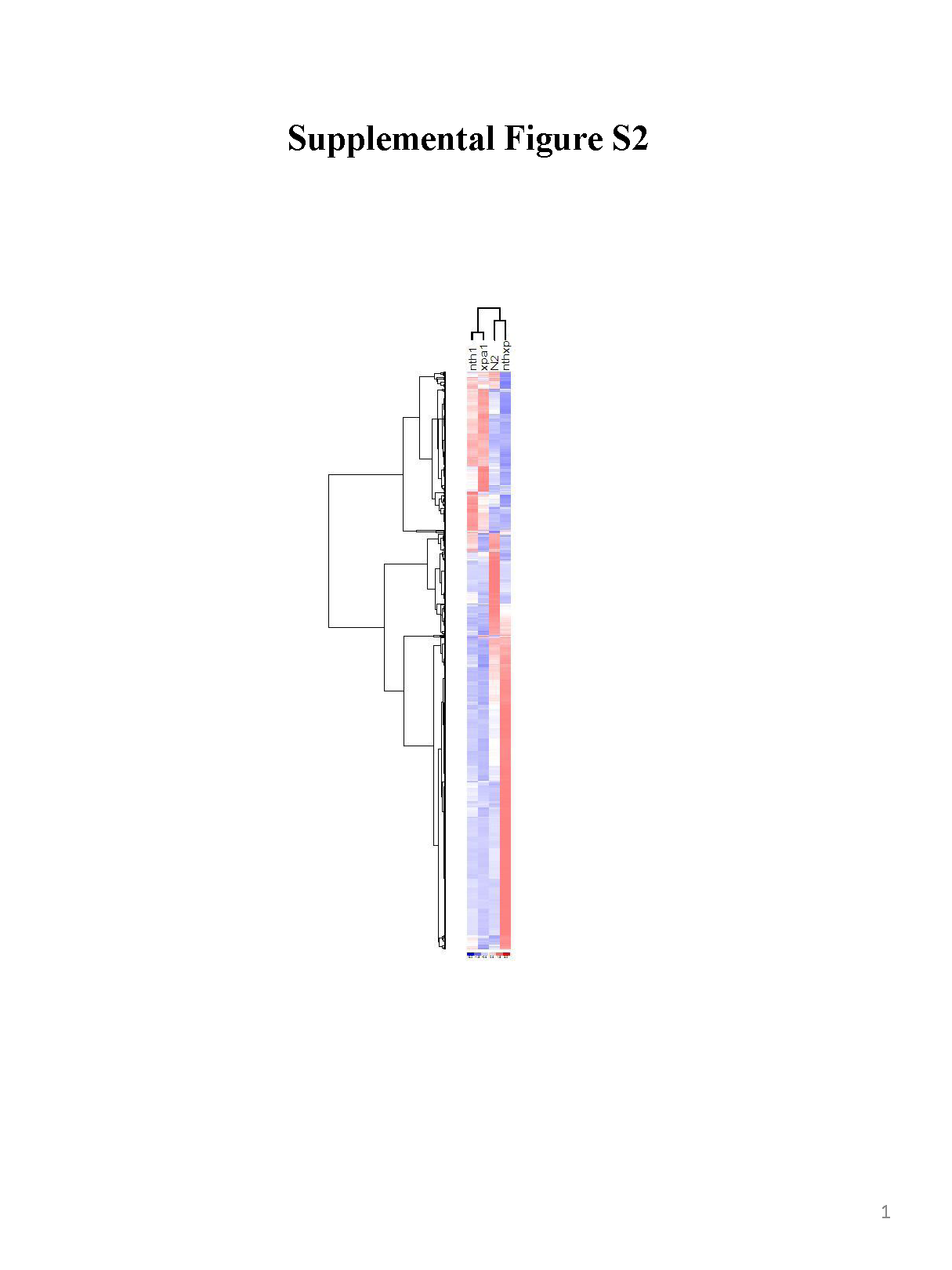

Supplement: Supplementary Figure 2 [file aging-02-133-s002.tif]
