# Supplementary material for: A Two-tiered compensatory response to loss of DNA repair modulates aging and stress response pathways
Source: Aging (Albany NY). 2010 Mar 29;2(3):133–59. doi: 10.18632/aging.100127 (PMC2871243; doi:10.18632/aging.100127)
Supplement: Supplementary Table 2 [file aging-02-133-s002.doc]

| **Genes** | **Description** | ***xpa*-*1*** | ***nth-1*** | ***aqp-1*** |
| --- | --- | --- | --- | --- |
| *ins-7* | insulin-like peptide | -8,80 | -3,00 | 1,99 |
| *lys-7* | an antimicrobial lysozyme | -3,36 | -3,40 | 2,09 |
| *hsp-12.6* | small heat shock protein | <1,8 (- regulated) | <1,8 (negatively regulated) | 2,16 |
| *spp-18* | SaPosin-like Protein family | -4,06 | -2,54 | 2,38 |
| *F46B6.8* | triglyceride lipase-cholesterol esterase | -3,34 | -3,43 | 2,43 |
| *T24C4.4* | unknown | -2,39 | <1,8 (similarly regulated) | 2,66 |
| *mtl-1* | copper-binding (detoxifying) metallothionein | -2,42 | -2,53 | 4,41 |

**Supplementary Table SII:** Overlapping genes in *aqp-1* and *nth-1* and *xpa-1.*

Genes found to be significantly differentially expressed in an *aqp-1* mutant, as analyzed in [40], were extracted and compared to transcripts in our microarray data set for *nth-1* and *xpa-1*. All 7 transcripts are regulated in a similar direction in the *xpa-1* mutant and 6 out of 7 in the *nth-1* mutant, as AQP-1 is upregulated here and absent in *aqp-1*.
